# Supplementary material for: Magnitude and associated factors of poor medication adherence among diabetic and hypertensive patients visiting public health facilities in Ethiopia during the COVID-19 pandemic
Source: PLoS One. 2021 Apr 6;16(4):e0249222. doi: 10.1371/journal.pone.0249222 (PMC8023457; doi:10.1371/journal.pone.0249222)
Supplement: S1 File — (DOCX) [file pone.0249222.s001.docx]

- 1. **Annex III: Data collection instruments (English)**

**Please encircle, fill or tick your responses in front of the questions presented below.**

**Part I: Socio demographic information**

| Q NO | Questionnaire | Coding categories/alternative |
| --- | --- | --- |
| 101 | Sex | Male  Female |
| 102 | Age | Age in year--------------- |
| 103 | Educational status | --------------------------- |
| 104 | Job | 1 Merchant  2 Civil servant  3 Private employed  4 student  5 house wife  6 jobless  7 other specify----------------------------- |
| 105 | Marital status | 1 single  2 married  3 separated  4 Divorced  5 Windowed |
| 106 | Religion | 1 Orthodox Christian  2 Protestant Christian  3 catholic  4 Muslim  5 other specify------------------------- |
| 107 | Numbers of family in the house | ----------- |
| 108 | Monthly income | In birr…………………… |

**Part II: Clinical and clinical related factors**

| Q .no | Questionnaire | Response |
| --- | --- | --- |
| 201 | **Attending clinic (main)** | **Other------Hypertension/DM------** |
| 202 | Duration of your illness? | Duration in year(s)…………… |
| 203 | How many chronic illnesses are you currently diagnosed with? | 1  2  3 or more |
| 204 | What kind of chronic illness do you have? | 1 Diabetes  2 Renal disease  3 Liver disease  4 HIV infection  5 Epilepsy  6 Hypertension  7other specify |
| 205 | Is there any sleep disturbance? | 1. Yes 2. No |
| 206 | Could you work your job properly? | 1. Yes 2. No |

**Part III: social factors**

| Q.no | Questionnaire | Categories/alternative |
| --- | --- | --- |
| 301 | How money people are you so close to that you can count on them if you have great personal problem? | 1 none  2 1-2  3 3-5  4 5+ |
| 302 | How much interest and concern do people show in what you do? | 1 none  2 little  3 uncertain  4 some  5 A lot |
| 303 | How easy is it to get practical help from others if you should need it? | 1 very difficult  2 difficult  3 possible  4 easy |

**Part Iv; substance use related questionnaire**

| Q.401 | In your life ,which of the following substances have you ever used ?(non-medical use only) | YES | NO |
| --- | --- | --- | --- |
|  | A. Tobacco products(cigarettes, etc) |  |  |
|  | B. Alcoholics beverages(beer,wine,etc) |  |  |
|  | C. Amphetamine type stimulants(khat chewing) |  |  |
|  | D. Other specify……………………… |  |  |
| Q.402 | Currently and in the last three months, which of the following substances have you ever used ?(nonmedical use only) |  |  |
|  | A. Tobacco products(cigarettes, etc) |  |  |
|  | B. Alcoholics beverages(beer, wine, etc) |  |  |
|  | C. Amphetamine type stimulants(khat) |  |  |
|  | D. Other specify……………………… |  |  |

**Part V:** **Medication adherence scale**

| Questions (adopted from Morisky medication adherence 8-scale [82] | Possible answers | | |
| --- | --- | --- | --- |
|  | Yes | No | |
| 501. Do you sometimes forget to take your medications? |  |  | |
| 702. Over the past 2 weeks, were there any days when you did not take your medicine? |  |  | |
| 503. Have you ever cut back or stopped taking your medication without telling your doctor because you felt worse when you took it? |  |  | |
| 504. When you travel or leave home, do you sometimes forget to bring along your medications? |  |  | |
| 505. Did you take your medicine yesterday? |  |  | |
| 506. When you feel like your illness is under control, do you sometimes stop taking your medicine? |  |  | |
| 507. Taking medication every day is a real inconvenience for some people. Do you ever feel hassled about sticking to your treatment plan? |  |  | |
| 508. How often do you have difficulty remembering to take all your medication? (select one below)  Never----------rarely--------not sure ------often ------Always ------- | | | |
| **Questions related to the perceived impact of COVID-19** | yes | | No |
| 509. Do you believe that the COVID-19 has negatively affected meeting your follow-up appointments? |  | |  |
| 510. Do you think that the COVID-19 has influenced availability of your chronic care medications? |  | |  |
| 511. Do you perceive that the COVID-19 has reduced the affordability /increased the price of anti-diabetic or anti-hypertensive medications? |  | |  |

Thank you

Name of data collector--------------- Name of supervisor-----------------------

sig. & date -------------- sig. & date ---------------------
