# Supplementary material for: Magnitude and associated factors of poor medication adherence among diabetic and hypertensive patients visiting public health facilities in Ethiopia during the COVID-19 pandemic
Source: PLoS One. 2021 Apr 6;16(4):e0249222. doi: 10.1371/journal.pone.0249222 (PMC8023457; doi:10.1371/journal.pone.0249222)
Supplement: S2 File — (DOCX) [file pone.0249222.s002.docx]

**የመረጃ መሰብሰቢያ መጠይቅ**

**ክፍል 1፤ ስነ-ህዝባዊ መረጃ**

| ተ.ቄ | መጠይቅ | አማራጭ ምላሽ |
| --- | --- | --- |
| 101 | ጾታ | 1.ወንድ  2. ሴት |
| 102 | እድሜ | ----------ዓመት |
| 103 | የትምህርት ሁኔታ | 1. ማንበብና መጻፍ የማይችል 2. ማንበብና መጻፍ የሚችል 3. መጀመሪያ ደረጃ (1-8ኛ ክፍል) 4. ሁለተኛ ደረጃ (9-12ኛ ከፍል) 5. ሰርቲፈኬት/ዲፕሎማ 6. ዲግሪና በላይ |
| 104 | ስራ | 1. ነጋደ----- 2. የመንገስት ሰራተኛ 3. የግል ሰራተኛ 4. ተማሪ 5. የቤት እመቤት 6. ስራ የሌለው 7. ሌላ--------------------------------------- |
| 105 | የትዳር ሁኔታ | 1. ያላገባ/ች 2. ያገባ/ች 3. የተለያዩ/አብሮ የማይኖሩ 4. የተፋቱ 5. የሞተባት/ችበት |
| 106 | ሀይማኖት | 1. ኦርቶዶክስ 2. ፕሮቴስታንት 3. ካቶሊክ 4. እስልምና 5. ሌላ------------------ |
| 107 | የቤተሰብ አባላት ብዛት | ------------------------- |
| 108 | ወርሀዊ ገቢ መጠን | --------ብር |

**ክፍል 2፡ የጤና ሁኔታ መረጃ**

| **ተ.ቁ** | **መጠይቅ** | አማራጪ ምላሾች |
| --- | --- | --- |
| 201 | የመጡበት ዋና የህክምና ክሊኒክ/አገልግሎት | 1/ኤ.አር.ቲ ክትትል  2/ የግፊት/ስኳር ክትትል |
| 202 | በሽታው ከጀመረዎት ምን ያህል ጊዜ ሆነው | --------ዓመት |
| 203 | መድሀኒት ከጀመሩ ምን ያህል ጊዜ ሆነዎት | --------ዓመት |
| 204 | ምን ያህል በተከታታይ የሚታከሟቸው ህመሞች አሉብዎት | 1. 1 2. 2 3. 3 ወይም ከዛ በላይ |
| 205 | ከተጠቀሱት ውስጥ የትኞቹ የክትትል ህመሞች አሉብዎት | 1.ስኳር  2. የኩላሊት ህመም  3. የጉበት ህመም  4. ኤች.አይ.ቪ  5. የሚጥል በሽታ  6. የደም ግፊት  7. ሌላ------------- |
| 206 | የእንቅልፍ ችግር አለብዎት | 1.አዎ  2.አይ |
| 207 | ስራዎትን በተገቢው ሁኔታ ማከናወን ይችላሉ | 1.አዎ  2.አይ |

**ክፍል 3፡ ማህበራዊ ጉዳዮች**

| ተ.ቁ | መጠይቅ | አማራጮች |
| --- | --- | --- |
| 301 | በችግር ጊዜ የሚያማክሯቸው ወይም ሊረዱዎት የሚችሉ ስንት ሰዎች አሉ | 1. ምንም 2. 1 3. 1-2 4. 3-4 5. 5 እና በላይ |
| 302 | ሌሎች ሰዎች ለእርስዎ ምን ዓይነት አመለካከት አላቸው | 1. አዎንታዊ 2. አሉታዊ 3. እርግጠኛ አይደለሁም |
| 303 | የሌሎች እርዳታ ቢያስፈልግዎ ለማግኘት ምን ያህል ቀላል ነው | 1. በጣም ከባድ 2. ከባድ 3. ቀላል 4. በጣም ቀላል |

**ክፍል 4፡ ሱስ አስያዥ መድሀኒቶችን የሚመለከት መጠይቅ**

| ተ.ቁ | መጠይቅ | አማራጮች | |
| --- | --- | --- | --- |
| 401 | በህይዎትዎ ከሚከተሉት መድሀኒቶች የትኞቹን ተጠቅመው ያውቃሉ | አዎ | አይ |
|  |  |  |  |
|  | ሀ/ የትንባሆ ውጤቶች (ሲጋራ፤) |  |  |
|  | ለ/ የአልኮል መጠጦች (ቢራ፤ወይን) |  |  |
|  | ሐ/ ጫት መቃም |  |  |
|  | መ/ ሌላ---------------------------------------- |  |  |
| 402 | በአሁኑ ሰዓትና ባለፉት ሶስት ወራት ከሚከተሉት የትኞቹን ተጠቅመው ያውቃሉ፤ | አዎ | አይ |
|  | ሀ/ የትንባሆ ውጤቶች (ሲጋራ፤) |  |  |
|  | ለ/ የአልኮል መጠጦች (ቢራ፤ወይን) |  |  |
|  | ሐ/ ጫት መቃም |  |  |
|  | መ/ ሌላ---------------------------------------- |  |  |

**ክፍል 5፤ የመድሀኒት ከትትልን የተመለከቱ መጠይቆች**

| ተ.ቁ | **መጠይቆች** | ምላሽ | | |
| --- | --- | --- | --- | --- |
|  |  | አዎ | አይ | |
| 501 | አንዳንድ ጊዜ መድሐኒትዎን የመርሳት ችግር ይገጥመዎታል |  |  | |
| 502 | ባለፉት 2 ሳምንታት መድሀኒትዎን ያልወሱባቸው ቀናት ነበሩ |  |  | |
| 503 | መድሀኒት ሲወስዱ የበለጠ ህመም ስለተሰማዎት መድሀኒቱን መውሰድ አቁመው ያውቃሉ |  |  | |
| 504 | ጉዞ ሲያደርጉ ወይም ከቤት ሲወጡ መድሀኒት ረስተው ያውቃሉ |  |  | |
| 505 | ትናንትና መድሀኒትዎን ወስደዋል |  |  | |
| 506 | ህመምዎ ሲሻልዎ ወይም የተቆጣጠሩት ሲመስልዎ መድሀኒትዎን ያቋርጣሉ |  |  | |
| 507 | ሁልጊዜ መድሀኒት መውሰድ ለአንዳንድ ሰዎች ከባድ ነው፤ ከአወሳሰድ ጋር በተያያዘ ህክምናዎን ለማቆም አስበው ያውቃሉ፤ |  |  | |
| 508 | መድሀኒትዎን በአግባቡ ለመውሰድ ምን ያህል ተቸግረው ያውቃሉ  በፍጹም------አልፎ አልፎ------እርግጠኛ አይደለሁም-----ብዙ ጊዜ----ሁልጊዜ------- | | | |
| **ከኮሮና ቫይረስ ጋር በተያያዘ** | | አዎ | | አይ |
| 509 | የኮሮና ቫይረስ ወረርሽኝ የህክምና ክትትልዎት ላይ አሉታዊ ተጽዕኖ አሳድሯል ብለው ያምናሉ፤ |  | |  |
| 510 | የኮሮና ቫይረስ ወረርሽኝ የክትትል መድሀኒትዎ አቅርቦት ላይ እጥረት/ ተጽዕኖ አድርጓል ብለው ያምናሉ፤ |  | |  |
| 511 | የኮሮና ቫይረስ ወረርሽኝ የስኳር/ግፊት መድሀኒት ዋጋ መጨመር ላይ ተጽዕኖ አርጓል ብለው ያምናሉ፤ |  | |  |

አመሰግናለሁ፡፡

የመረጃ ሰብሳቢው ስም------------------------- የሱፐርቫይዘር ስም---------------------

ፊርማ ------------------------- ፊርማ -----------------
