# Supplementary material for: Magnitude and associated factors of poor medication adherence among diabetic and hypertensive patients visiting public health facilities in Ethiopia during the COVID-19 pandemic
Source: PLoS One. 2021 Apr 6;16(4):e0249222. doi: 10.1371/journal.pone.0249222 (PMC8023457; doi:10.1371/journal.pone.0249222)
Supplement: Attachment — Submitted filename: Response to reviewers.docx [file pone.0249222.s003.docx]

**Response to reviewers**

# Thank you for the email, and requesting us to make revision on our manuscript titled, “Magnitude and associated factors to medication poor adherence among diabetic and hypertensive patients visiting Public health facilities in Ethiopia during the Coronavirus pandemic” submitted for potential publication in your journal.  The comments forwarded were very insightful, and constructive. Each point raised both by the academic editor and reviewer (s) have been thoroughly looked into and addressed accordingly as presented below.  Answers to academic editor comments:

# 1. Please ensure that your manuscript meets PLOS ONE's style requirements, including those for file naming.

*Ans. Changes have been made on figure and table labels as well as captions by referring to the attached web ling.*

2. Please include additional information regarding the survey or questionnaire used in the study and ensure that you have provided sufficient details that others could replicate the analyses. For instance, if you developed a questionnaire as part of this study and it is not under a copyright more restrictive than CC-BY, please include a copy, in both the original language and English, as Supporting Information.
Furthermore, within the Methods section, please provide a description of how participants were recruited for the study.

*Ans. A copy of the data collection instrument both in English, and Amharic has been attached as a supplementary file. Further, revision regarding how participants were recruited has been included under the ‘sampling technique/method’ sub-section.*

3. We suggest you thoroughly copyedit your manuscript for language usage, spelling, and grammar.

*Ans. A revision has been made in light with spelling, grammar usage and tenses. We employed the research team, and online free language editing services to fix such problems.*

4. Please include in your Methods section (or in Supplementary Information files) the participating hospitals/institutions. Furthermore, please provide additional clarifications as to whether IRB of all participating institutions approved the study prior to data collection.

*Ans. A description of all (seven) participating hospitals and health centers has been included under ‘study settings, design and period’ sub-section. The IRBs that approved the study have also been mentioned under the ‘Ethical approval’ part.*

*5.* Please ensure that you include a title page within your main document.

*Ans. Title page included within the main manuscript file.*

6. Your ethics statement should only appear in the Methods section of your manuscript. If your ethics statement is written in any section besides the Methods, please move it to the Methods section and delete it from any other section.

*Ans. The ethics statement is presented in the methods section.*

**Answers to reviewer #1 comments**

Abstract: the abstract is well written but quite long. Please be concise.

*Ans. Abstract has been revised accordingly.*

Introduction: THe introduction reads as a textbook with little conffection between the paragrafs.
The first paragraph starts with defining the WHO criteria for adherence. What does adherence refer to. Such a paragfar is not appealing to read further. I suggest to start with why drug adherence for hyperntension and diabetes is of interenst to study. he paragraphs discussing the literature may be more suitable for the discussion (paragraf 3 and 4). Could be in 2 or 3 sentecences in the introduction.

*Ans. We have revised the introduction accordingly. The introduction has been started with a statement of’ why adherence to medication is important in the management of chronic illnesses. Further changes are made in the middle 3 and 4 paragraphs based on the comments from both reviewers.*

Methods:
"A cross sectional study design was conducted in August 2020 among seven public health facilities (two hospitals and five health centers) to assess adherence of patients to antidiabetic and antihypertensive medications during the COVID-19
pandemic. " Was it from the start of august till the end of august? Wat was the time window for the study? Who assessed the data? Wat is there an approval from the Medical ethical commitee?
Where only chronic patients included or also patients with hypertension and DM de novo.

*Ans. This statement has been revised, and presented with clear timeline, duration from 1 through 30 of August 2020. The change is updated in the methods and abstract sections. Ethical approval and support letter was sought at two institutions. This has been updated in the methods section. Patients with at least 6 months of follow-up before the start of the data collection period have been included.*

"Content validity was of the tool was ensured by study team. Cronbach’s
alpha test of reliability of the scale among pretested samples showed acceptable range for both
hospitals (α=0.88) and health centers [α=0.63]. "This is describtion of the results. I suggest to transfer it to the results section.

*Ans. This part has been presented at the end of the ‘profiles of participants’*

Results:
Please put tables at the end of the paper.
The description of the results is not consistent. For example: AOR: 0.58; 95%C.I: 0.35-0.97 and AOR: 0.50; 95% C.I:0.27-0.89

*Ans. Tables have been shifted to the end of the paper. Also, consistency to report both OR and percentages was endured throughout the paper.*

Discussion:
Can be extended with the literature discussion from the introduction.
Please start the discussion with a short description of the results.
The conclusion is again a summary of the results, please make it a conclusion and add a clear recommendation paragraph.
Please add in the discussion a clear strength and limitation paragraph.

*Ans. Literature earlier included in the introduction has, now, been shifted at the discussion part. The discussion part is presented with a deductive approach often a recommended in quantitative research. The conclusion part has been revised and rephrased. A separate paragraph (last) on strength and limitation is provided.*

***Answers to reviewer # comments***

**Introduction**: good summary. However, the authors could highlight a bit more what the added value of their work is, in addition to investigating the situation during the COVID pandemia, as compared to the papers cited in the introduction (e.g. differences in the setting as I can imaging that the populations seen in different settings differ, or why numbers of other studies may be not representative, or that the population of this study is the same as that of one of the other studies, allowing to investigate the direct impact of COVID on adherence).

*Ans. The introduction has been revised based on the comment given here and reviewer 1. More essence to this comment was added at the line 1-3 of the last paragraph. This covers on; potential variation between the current and existing evidence on the topic and study setting.*

Methods:
Where all patients including during the time period or was it a selected group of patients? Did all patients consent to participate in the study? Probably not, To what extend did those not included differ from those that participated in this study? It may be helpful to have a figure explaining the patient flow.
The sample size calculation is unclear. What was the aim / target for the calculation? Please be more precise. I do not understand it at present.
It would be helpful to add the questionnaire, at least as supplementary file. This makes it also easier to understand exactly how good adherence was defined.
Is it correct that participating patients did not give written informed consent? If yes, was this an exemption specifically approved by the Ethical Committee a priori?
Were all variables included in multivariable analysis or were there any selection criteria? If no selection criteria applied, the power may be not sufficient for multivariable analysis given the number of independent variables. In this case, I would recommend to first perform a univariable analysis and only include those with a e.g. P<0.1 in the multivariable analysis. At present, it is not clear why some variables are mentioned in table 2 and others not.

*Ans. Considering the COVID-19 pandemic peak spread time (July to August) in the study setting which also have affected the usual patient flow, a consecutive sampling method was made. The potential variation that is inevitable among the patients included and those not included has been admitted and mentioned as the possible limitation of the study. Yet, a few number of patients (n=13) were missing. This however, is above the minimum sample size estimated presented in the methods part. Hence, the minimum sample size was calculated using the single population formula. Attaining, 409 was then considered adequate. As the inclusion in the study was deemed to cause a less than minimum risk, only a verbal consent was requested, and the respective ethics committees have approved the study. inclusion of multivariable analysis was made based a P<=0.2 in a bi-variable analysis. Detailed revisions are included in the methods section.*

Results:
How was the presence of close people defined? How was co-morbidity defined? Would it not be better to name the prevalence of co-morbidities (at least for the most prevalent ones)? In addition, it would be interesting with which medication patients were treated.
Figure 1. % instead of absolute numbers would be better.
As far as I understand, an OR of <1.0 indicates less non-adherence, i.e. better adherence. I think that it is easier to understand if you only better adherence (with an OR > 1) and worse adherence (with an OR <1). This is easier to understand. This obviously also applies to the according parts of the discussion (e.g. association with better adherence instead of association with lower odds of poor adherence).
Would it not be better to use the poverty line as cut-off instead of the median within the study, particularly as this is also related to the discussion?
Please define AOR and COR in table 2.

*Ans. An additional operational definition has been placed for ‘close people’ and ‘comorbidity’ under the ‘operational definition’ section. A description for prevalent comorbid conditions has been presented under the results section. Percent values were substituted for Fig1 as per the recommendation. The Odds ratio (OR) presentation and further revision (such as, education level turned out to be a non-significant predictor ) were made to the last table. “lower odds of poor adherence’ was changed [rephrased] to ‘higher odds of good adherence. However, the figures were retained in their original values as outcome of interest in this study was ‘poor medication adherence’. A revision to the classification of income level was made based on the poverty line report of the World Bank. AOR and COR were defined in table 2.*

Discussion:
It is a bit confusing that you refer that 40% are negatively affected by the pandemia regarding non-adherence, but in fact, there were 57%. This applies also to the abstract. This is confusing. In addition, you do not need to repeat results in the discussion (use e.g. the majority instead).
Please discuss your findings in the light of previous findings in your country. You only do so regarding the overall adherence. It would be interesting to hear if factors associated with better adherence were similar in other studies and what potential explanations for the differences are.
The finding and discussion of low income being associated with better adherence is interesting. What do other studies say on this and what may be the differences with your findings? This is missing. The findings related to co-morbidites etc. are in line with your findings. This needs to be mention and is separate to the discussion about impact of low income.
Have a separate paragraph regarding limitations. You also need to add that the number of included patients is limited and other important factors may have been missed because of this limitation. In addition, the selection of patients may be a limitation (not only that inclusion was limited to one month), but this is difficult to judge as nothing is reported in this regard (see above).
Minor comments:
There are quite some spelling and grammatical mistakes (e.g. incorrect use of tenses). Please check and correct.

*Ans. A revision has been made for clarity per reviewer’s comment. A 40% believed on the COVID-19 impact on medication availability and follow-up appointments, and 57% any of the 3 aspects. Previous studies in Ethiopia have been looked in to and compared, especially, on common risk factors reported so far. Unfortunately, there was no report that accords with effect of low income in the present setting. That’s why paragraph 5 starts by stating’ In paradox with the popular belief [40, 45], lower income was found to be associated with higher odds of good adherence to antidiabetic and antihypertensive medications. A separate paragraph (paragraph 5) was presented to talk on low income and others (paragraph 6). Comment on potential limitation of the study [missed and small sample …) has been included in the last paragraph. As possible, we have made revision on the language of the manuscript.*
